# Supplementary material for: A Dual-Mode Method Based on Aptamer Recognition and Time-Resolved Fluorescence Resonance Energy Transfer for Histamine Detection in Fish
Source: Molecules. 2022 Dec 9;27(24):8711. doi: 10.3390/molecules27248711 (PMC9785670; doi:10.3390/molecules27248711)
Supplement: Supplementary file 1 [file molecules-27-08711-s001.zip › molecules-2053844-supplementary.pdf]

Supporting Information for a dual-mode method based on aptamer recognition and time-resolved fluo-rescence resonance energy transfer for the detection of histamine in fish

Xin Wang [1†], Fu Yang [1†], Chengfang Deng [1], Yujie Zhang [1], Xiao Yang [1,2], Xianggui Chen [1,2], Yukun Huang [1,2*], Hua Ye [3*], Jianjun Zhong [3], Zhouping Wang [4]

1 School of Food and Biological Engineering, Xihua University, Chengdu 610039, China
2 Chongqing Key Laboratory of Speciality Food Co-Built by Sichuan and Chongqing, Chengdu 610039, China
3 School of Grain Science and Technology, Jiangsu University of Science and Technology, Zhenjiang 212100, China
4 School of Food Science and Technology, Jiangnan University; Wuxi 214122, China
* Correspondence: *Y. Huang*, hyk_diana@163.com; *H. Ye*, huaye@just.edu.cn.
† These authors contributed equally to this work.

List of contents

S1 Supplementary materials 1
Figure S1. The prepared AuNPs object. 1
Figure S2. (a) Ultraviolet peak spectrum of AuNPs; (b) Electron microscopic picture of AuNPs 1
Figure S3. (a) Time-resolved fluorescence spectra; (b) Time-resolved fluorescence nanoparticle electron microscope 2
Supplementary Material for HPLC method operation for Sample Analysis and Detection 2

S1 Supplementary materials

1) Preparation of supplementary materials for gold nanoparticles (AuNPs)

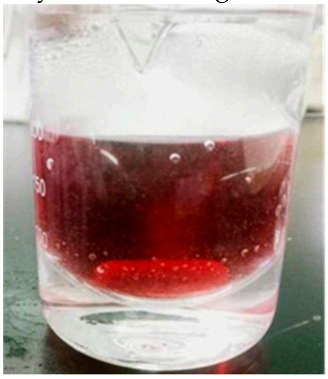

Figure S1. The prepared AuNPs object.

Figure S1 shows the experimentally prepared AuNPs solution, which was characterized, and Figure S2 (a) shows the UV-Vis spectrum of the AuNPs solution, which was scanned at 400 to 800 nm with a maximum absorption wavelength of 523 nm. transmission electron microscopy photographs show that the morphology of the synthesized AuNPs is essentially spherical and uniformly dispersed (Figure S2 (b)).

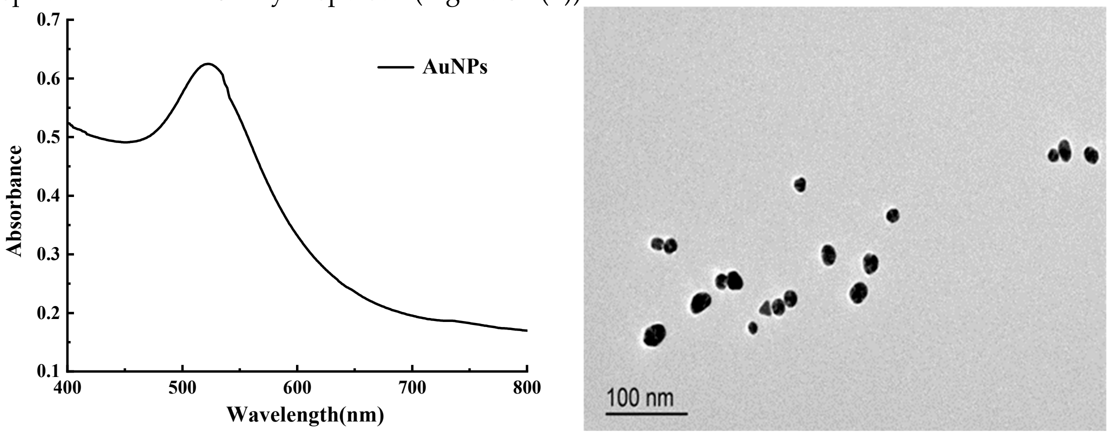

(**a**) (**b**)

Figure S2. (**a**) Ultraviolet peak spectrum of AuNPs; (**b**) Electron microscopic picture of AuNPs.

2) Preparation of supplementary materials for NaYF4:Ce/Tb nanofluorescent material

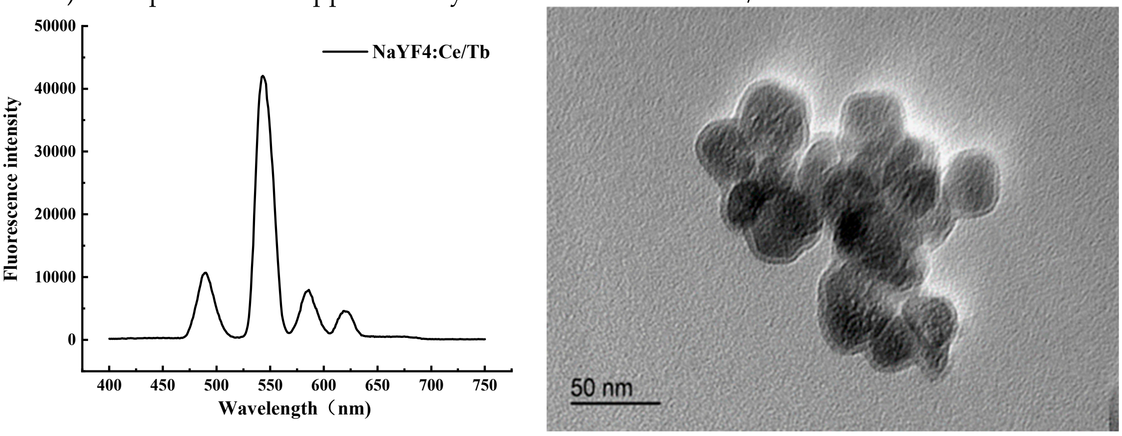

(**a**) (**b**)

Figure S3. (**a**) Time-resolved fluorescence spectra; (**b**) Time-resolved fluorescence nanoparticle electron microscope.

Figure 3 (a) shows the time-resolved fluorescence spectra of the NaYF4:Ce/Tb nanofluorescent material, with the emission at 490 nm, 545 nm, 587 nm and 621 nm without overlapping features, relatively high fluorescence intensity, large separation and no mutual interference between them, suitable for the determination of the target. Figure 3 (b) shows the time-resolved fluorescence transmission electron micrograph of the nanofluorescent material, which shows that the morphology of NaYF4:Ce/Tb nanofluorescent material is basically spherical and in the aggregated state.

3) Supplementary material for histamine detection by fluorescence and colorimetric methods

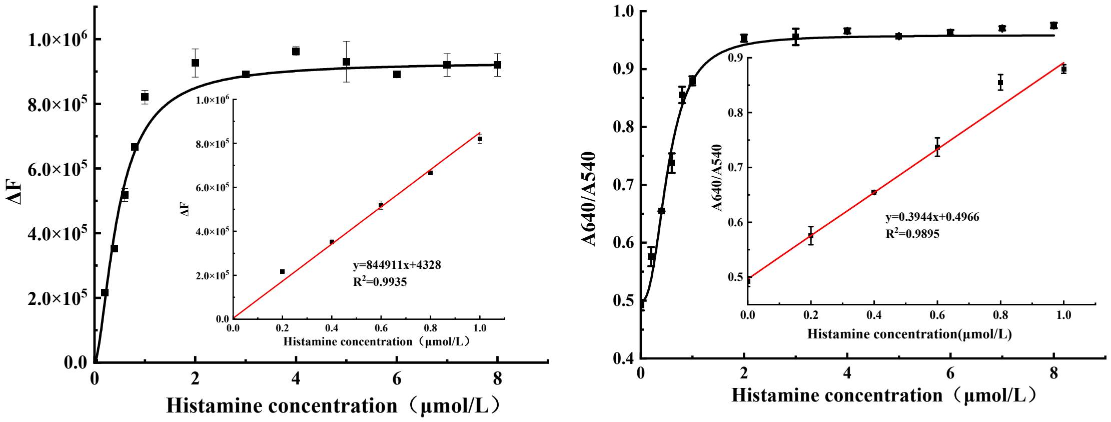

(**a**) (**b**)

Figure S4. (**a**) Relationship between fluorescence recovery value (ΔF) and histamine concentration; (**b**) Absorbance ratio (A_{640}/A_{540}) and histamine concentration;

Figures S4(a) and 4(b) show the relationship between the fluorescence and colorimetric methods for the detection of histamine, respectively. The histamine concentration in the range of 0.2-1.0 μmol/L showed a good linear relationship with the fluorescence recovery value with the correlation coefficient of R^2=0.9935 and the linear regression equation of Y=844911X+4328. The detection limit of 9. 21 nmol/L can be calculated from 3σ/K (where σ is the blank K is the slope of the calibration curve); the absorbance ratio (A640/A540) also showed a good linear relationship with the histamine concentration in the range of 0.2-1.0 μmol/L with the correlation coefficient of R^2=0.9895, the linear regression equation of Y=0.3944X+0.4966, and the detection limit of 69.37 nmol/L.

4) Supplementary Material for HPLC method operation for Sample Analysis and Detection

After the preparation of the sample, accurately weigh 10 g of minced or homogenized aquatic products, put them in a conical flask with a stopper, add the internal standard solution, fully mix them with the sample, and add 20 ml trichloroacetic acid solution for shaking extraction. Adding sodium chloride, vortex oscillating until sodium chloride is completely dissolved, adding n-hexane to remove fat, and vortex oscillating. The sample solution after fat removal is plugged into a centrifuge tube, and the pH is adjusted to about 12.0 with 5 mol/L sodium hydroxide solution. Add the mixed solution of n-butanol/chloroform (1+1) and swirl. Add 5 ml hydrochloric acid (1 mol/L) into the extract, mix well, blow-dry with nitrogen in a water bath at 40℃ and swirl the hydrochloric acid to completely dissolve the residue before derivatization.

Add saturated sodium bicarbonate solution, sodium hydroxide solution and derivatization reagent to the sample solution to be derivatized in sequence, vortex and mix well, then put it in a constant temperature water bath at 60℃ for derivatization for 15 min, take it out, add sodium glutamate solution separately, shake and mix well, and react at 0℃ for

15 min. Take it out, cool it to room temperature, add 1 ml of water, mix with vortex, remove acetone by nitrogen blowing in 40℃ water bath, add sodium chloride, vortex and shake until sodium chloride is completely dissolved, then add ether, vortex and shake for 2 min, stand for layering, suck out the upper organic phase (ether layer), extract again, combine ether extracts, and blow dry with nitrogen in 0℃ water bath. Adding nitrile, swirling and shaking to completely dissolve the residue, and filtering with a filter membrane needle filter in the sample inlet vial to be determined. Derivation of vertebral standard is the same as above.
